# Improving delirium screening and recognition in UK hospitals: results of a multi-centre quality improvement project

**DOI:** 10.1093/ageing/afab243

**Published:** 2022-02-25

**Authors:** Geriatric Medicine Research Collaborative

**Affiliations:** Institute of Inflammation and Ageing, College of Medical and Dental Sciences, University of Birmingham, Birmingham B15 2TT, UK

**Keywords:** delirium, quality improvement, collaborative, education, older people

## Abstract

**Background:**

delirium is an acute severe neuropsychiatric condition associated with adverse outcomes, particularly in older adults. However, it is frequently under-recognised.

**Methods:**

this multi-centre quality improvement project utilised a collaborative approach to implementation of changes at sites, with the aim to improve delirium screening, recognition and documentation on discharge summaries. Resources, including delirium guidelines and presentations, were shared between sites, and broad details of local interventions were collected. Three timepoints of data collection (14 March 2018, 14 September 2018 and 13 March 2019) were conducted to assess screening, recognition and documentation of delirium in unscheduled admissions of adults aged ≥65 years old. The impact of local interventions and site-specific factors was assessed using logistic regression analysis, adjusting for patient factors.

**Results:**

a total of 3,013 patients (mean age 80.2, 53.8% females) were recruited across the three timepoints. Screening for delirium was associated with increased odds of recognition (aOR 4.75, CI 2.98–7.56; *P* < 0.001); this was not affected by grade/profession of screener. Rates of screening, recognition and discharge documentation improved across the three timepoints of data collection. The presence of a local delirium specialist team was associated with increased rates of screening for delirium (aOR 1.75, CI 1.41–2.18; *P* < 0.001), and the presence of a geriatric medicine team embedded into the admissions unit was associated with increased recognition rates (aOR 1.78, CI 1.09–2.92; *P* = 0.022).

**Conclusion:**

delirium screening is associated with improved recognition. Interventions that strive to improve screening within a culture of delirium awareness are encouraged.

## Key Points

- A multi-centre ‘crowdsourcing’ approach to quality improvement is feasible.
- Delirium screening, recognition and discharge documentation improved across each round of data collection.
- Screening for delirium increases the odds of recognition, and drives to improve screening are recommended.

## Background

Delirium is an acute neuropsychiatric state defined by cognitive change and altered consciousness that occurs secondary to physical precipitants, particularly in older adults during hospitalisation [1]. Delirium is associated with adverse outcomes, but is frequently under-recognised [2]. Our previous research demonstrated that delirium screening increases recognition [1]. However, delirium screening was inconsistent. Screening and recognition were particularly reduced in surgical specialties [1]. Although delirium is reversible, it is associated with increased risk of later life dementia diagnosis [3]. Follow-up of patients who have experienced delirium is, therefore, vital. Unfortunately, delirium documentation on discharge summaries is infrequently performed [4]. In the UK, National Institute for Health and Care Excellence guidelines recommend that all adults aged ≥65 years old are screened for delirium on admission to hospital [5], and that delirium diagnoses are communicated to General Practitioners (GPs) on hospital discharge [6].

## Aims

- To improve screening and recognition of delirium in older adults admitted to acute care hospitals as unscheduled admissions.
- To improve documentation of delirium diagnoses on discharge summaries.

## Methods

This study presents results of a multi-centre quality improvement project, utilising a collaborative approach. Resources and knowledge were shared between sites, and sites were able to implement interventions locally according to service needs. Three timepoints of data collection were utilised (14 March 2018, 14 September 2018 and 13 March 2019). All timepoints of data collection included newly admitted (unscheduled) patients aged ≥65 years old to acute care trusts (all specialties, excluding critical care). We assessed for statistical significance of differences in likelihood of screening, recognition and discharge documentation across timepoints using multivariable logistic regression analysis. Full methodology is Supplementary data (Figure S1, Figure S2) are available in *Age and Ageing* online.

## Results

Timepoint 1 included 1,507 patients from 44 sites, Timepoint 2 included 656 patients from 26 sites and Timepoint 3 included 850 patients from 48 sites. Eighty-two sites contributed data to at least one timepoint (Table S1, Supplementary data are available in *Age and Ageing* online). Overall prevalence of delirium across all timepoints was 16.3% (491/2,522). Characteristics of patients at each timepoint are shown in Table 1. Interventions implemented at sites between timepoints are shown in Table S2, Supplementary data (Figure S1, Figure S2) are available in *Age and Ageing* online.

**Table 1:** Characteristics of study population across all timepoints

		Timepoint 1		Timepoint 2		Timepoint 3	
		Delirium (*N* = 222)	No delirium (*N* = 1,285)	Delirium (*N* = 133)	No delirium (*N* = 523)	Delirium (*N* = 136)	No delirium (*N* = 714)
Age—mean (SD)		84.0 (7.4)	79.3 (8.3)	82.8 (8.1)	79.6 (8.2)	83.3 (7.9)	80.0 (8.5)
Gender—% females (*N*)		60.8 (135)	51.6 (663)	50.4 (67)	55.3 (289)	60.3 (82)	53.8 (384)
Dementia—% (*N*)		35.1 (78)	12.9 (166)	41.4 (55)	14.0 (73)	54.4 (74)	16.2 (116)
Clinical Frailty Scale	1	0.5 (1)	4.3 (55)	1.5 (2)	4.1 (21)	0	5.4 (39)
	2	2.7 (6)	11.3 (145)	1.5 (2)	10.9 (57)	2.2 (3)	9.7 (69)
	3	3.6 (8)	19.7 (253)	6.8 (9)	21.0 (110)	2.2 (3)	17.8 (127)
	4	10.8 (24)	18.1 (232)	15.0 (20)	19.5 (102)	8.1 (11)	16.0 (114)
	5	21.2 (47)	15.9 (204)	10.5 (14)	15.1 (79)	19.1 (26)	17.9 (128)
	6	28.4 (63)	17.6 (226)	30.8 (41)	15.9 (83)	29.4 (40)	17.4 (124)
	7	25.7 (57)	9.3 (119)	26.3 (35)	10.5 (55)	32.4 (44)	9.7 (69)
	8	4.5 (10)	0.9 (12)	6.0 (8)	1.5 (8)	5.1 (7)	1.3 (9)
	9	0 (0)	0.2 (3)	1.5 (2)	1.3 (7)	0 (0)	0.6 (4)
Specialty	Acute medicine	47.7 (106)	42.2 (542)	21.8 (29)	21.2 (111)	27.2 (37)	18.5 (132)
	Geriatric medicine	26.6 (59)	16.0 (206)	45.1 (60)	20.3 (106)	37.5 (51)	18.8 (134)
	Stroke	1.8 (4)	4.0 (52)	0.8 (1)	5.0 (26)	1.5 (2)	3.9 (28)
	Other medicine	14.0 (31)	22.1 (284)	21.1 (28)	27.9 (146)	19.1 (26)	35.9 (256)
	Orthopaedic surgery	6.8 (15)	6.2 (80)	6.0 (8)	9.0 (47)	8.1 (11)	9.0 (64)
	General surgery	3.2 (7)	6.5 (83)	4.5 (6)	8.0 (42)	4.4 (6)	9.1 (65)
	Other surgery	1.8 (4)	3.0 (38)	0.8 (1)	8.6 (45)	2.2 (3)	4.3 (31)

### Delirium screening

Delirium screening increased across timepoints (27.3% versus 29.6% versus 37.1%; *P* < 0.001). Odds of screening increased between Timepoints 1 and 3 (aOR 1.33, CI 1.08–1.65; *P* = 0.001; Table 2). Delirium screening was associated with increasing age, mild–moderate (but not severe) frailty and dementia. Odds of delirium screening were increased with presence of specialist delirium teams (aOR 1.75, CI 1.41–2.18; *P* < 0.001; Table S3, Supplementary data are available in *Age and Ageing* online).

**Table 2:** Logistic regression analysis for odds of screening, recognition and discharge documentation between timepoints

	**Timepoint 2 versus Timepoint 1**		**Timepoint 3 versus Timepoint 1**	
	**OR (CI)**	*P* **value**	**OR (CI)**	*P* **value**
**Delirium screening**				
Unadjusted	1.18 (0.95–1.46)	0.142	1.45 (1.18–1.78)	<0.001
Adjusted^¥^	1.07 (0.85–1.34)	0.570	1.33 (1.08–1.65)	0.001
**Delirium recognition**				
Unadjusted	2.60 (1.62–4.16)	<0.001	2.31 (1.41–3.77)	0.001
Adjusted^ǂ^	1.93 (1.11–3.35)	0.019	2.33 (1.31–4.15)	0.004
**Discharge documentation**				
Unadjusted	2.34 (1.23–4.46)	0.009	1.78 (1.02–3.11)	0.042
Adjusted^¥^	2.27 (1.10–4.68)	0.026	1.73 (0.93–3.24)	0.085

^¥^Adjusted for Clinical Frailty Scale, age, gender, dementia status, specialty and site-specific factors.

^ǂ^Adjusted for screening, subtype, Clinical Frailty Scale, age, gender, dementia status, specialty and site-specific factors.

### Delirium recognition

Delirium recognition increased across timepoints (34.2% versus 57.1% versus 63.2%; *P* < 0.001). Odds of recognition increased between Timepoints 1 and 2 (aOR 1.93, CI 1.11–3.35; *P* = 0.019), and 1 and 3 (aOR 2.33, CI 1.31–4.15; *P* = 0.004; Table 2). Screening for delirium was associated with delirium recognition (aOR 4.75, CI 2.98–7.56; *P* < 0.001); this was not affected by grade/profession of screener. Recognition odds were increased in patients with dementia (aOR 1.73, CI 1.06–2.84; *P* = 0.029), and presence of geriatric teams embedded into admissions units (aOR 1.78, CI 1.09–2.92; *P* = 0.022). Admissions under general (aOR 0.11, CI 0.02–0.58; *P* = 0.009) or orthopaedic (aOR 0.27, CI 0.09–0.79; *P* = 0.017) surgery were associated with reduced delirium recognition (Table S4, Supplementary data are available in *Age and Ageing* online).

### Discharge documentation

Discharge documentation increased across timepoints (28.6% versus 48.4% versus 46.6%; *P* = 0.002) (Table 2). Odds of discharge documentation increased from Timepoint 1 to 2 (aOR 2.34, CI 1.23–4.46; *P* = 0.009). Odds of delirium documentation were increased in patients with dementia (aOR 2.01, CI 1.16–3.48; *P* = 0.012), but not affected by site-specific factors (Table S5, Supplementary data are available in *Age and Ageing* online).

## Discussion

Delirium screening, recognition and discharge documentation all improved overall, demonstrating that rates of these are not fixed/inalterable. Importantly, screening is associated with increased odds of recognition; efforts to increase screening should be encouraged. Notably, grade and profession of screener did not affect recognition. Therefore, interventions to improve recognition may utilise trained multiprofessional screeners and junior staff. The 4AT has been validated for use by all healthcare professionals with minimal training [7, 8].

Discharge documentation was more likely in patients with pre-existing dementia. Reasons for this are unclear, but may relate to greater awareness of importance of delirium in dementia, in a similar manner to increased recognition rates. There may be a general misunderstanding as to why communication of delirium diagnoses to GPs is important, in terms of highlighting risk of future cognitive decline [3, 9]. Encouragingly, documentation improved across timepoints. Previous studies have shown that discharge documentation is often inadequate across many settings [10].

### External validity

Overall delirium prevalence was 16.3%. This is lower than a previous single-site point prevalence study (19.6%) [11]. Differences are accounted for by inclusion of incident delirium in the latter study; only prevalent cases at admission were included at Timepoints 1 and 3. A higher prevalence rate (22.9%) was reported in a study considering positive screen with 4AT alone and not reference-standard delirium diagnosis [12]. Previous single centre quality improvement projects have demonstrated similar improvements in delirium screening and recognition with local interventions. A previous study involving implementation of a local delirium pathway and multidisciplinary teaching programme demonstrated improvement in delirium recognition rates from 5.7 to 35% over 11 weeks [13]. Similarly, implementation of dedicated teaching sessions within an acute medical unit, and management bundle with checklists, resulted in improved delirium screening rates from 40 to 61% [14].

### Internal validity

Patient characteristics were similar across timepoints with regards to age, gender and specialties. Most patients were within acute and geriatric medicine specialties, which is consistent with recognised pathways of care within the UK [15]. However, at later timepoints higher dementia rates were recorded. Our results demonstrated that delirium was more likely to be recognised in patients with dementia. However, effects persisted in multivariable models adjusting for dementia.

We recognise that a significant limitation is that not all the same sites participated at each timepoint. Analyses and interpretation were performed for sites overall rather than at site level; site level analysis was not possible due to individually small numbers. Nevertheless, improvement in screening, recognition and documentation across timepoints demonstrates that such improvements are possible. We acknowledge that methodology differed between timepoints; data was collected prospectively at Timepoints 1 and 3 but retrospectively at Timepoint 2. We consider this variation unlikely to have significantly impacted upon results. Validated methodology for retrospective delirium ascertainment was used at Timepoint 2 [16, 17]. In addition, although this may have led to differences in recognition rates, this should not have affected screening rates. Documentation of screening was extracted from clinical records across all timepoints.

We cannot be certain specific interventions led to improvements, or if these relate to external factors. It is conceivable that improvements across timepoints related to improved culture of embedding delirium screening and assessment into clinical practice. This may have occurred due to leadership of collaborators at sites. However, many doctors rotated sites during this project, and collaborators at sites differed between timepoints. This suggests that changes can be sustained, even where leadership is rotated. Improved delirium screening and recognition rates may also be related to external factors. The International Federation of Delirium Studies (iDelirium) is an international collaboration of societies, which seeks to educate patients, caregivers, professionals and policy makers about delirium [18]. The society constantly aims to increase awareness of delirium, however, national campaigns peek around the time of WDAD (i.e. first and last timepoints) [18].

### Recommendations

Our approach of sharing guidelines, resources and a central data collection point is feasible in involving multiple centres across multiple timepoints. Methodology was sufficiently simple to enable healthcare professionals of any grade or profession to be involved with screening and data collection. This is a model that may be replicated in future collaborative quality improvement projects.

Despite the focus on screening and recognition, it is important to recognise that assessment is only part of management. If delirium is present and recognised this should prompt healthcare professionals to take action by identifying and treating underlying causes [19]. Education should focus on rationale behind actions, such as the need to ensure delirium is communicated to primary care to enable appropriate follow-up of cognitive trajectories. Delirium may take some time to fully resolve [20], and communication to primary care is of utmost importance.

Although it was not possible to analyse site level data for effectiveness of individual interventions at site, we were able to identify site factors that were predictive of screening and recognition. Specialist delirium teams were associated with improved screening rates, and geriatric medicine teams embedded into admissions units were associated with improved recognition rates. Where sites are seeking to improve screening and recognition rates locally, we suggest that these findings are considered in service development. There was a very strong association (nearly fivefold) between delirium screening and likelihood of delirium recognition. Thus, drivers towards increased delirium screening are likely to prove beneficial.

## Conclusions

A collaborative approach to multi-centre quality improvement is feasible; including multiple data collection timepoints, and sharing of guidelines/resources and knowledge across sites. Importantly, screening, recognition and delirium documentation rates are not fixed/unalterable; improved rates across timepoints suggest potential for responsiveness to interventions. Screening for delirium is associated with increased likelihood of delirium recognition. We encourage implementation of interventions to improve recognition through way of increased screening, alongside sustainable culture changes.

## Supplementary Material

aa-21-0029-File002_afab243

## Figures and Tables

**Table:** Members of the Geriatric Medicine Research Collaborative

**Manuscript preparation**			
**First name**	**Middle initial**	**Last name**	**Affiliation**
Julian		Lin	Torbay and South Devon NHS Foundation Trust
Sindhoora		Dama	South Warwickshire Foundation Trust
Carly		Welch	University of Birmingham
**Steering group**			
Thomas	A.	Jackson	University of Birmingham
Laura		Magill	
Lauren		McCluskey	
Rita		Perry	
Daisy		Wilson	
**Advisory group**			
Claire		Copeland	NHS Forth Valley
Emma		Cunningham	Queen’s University Belfast
Daniel		Davis	University College London
Jonathan		Treml	University Hospitals Birmingham NHS Foundation Trust
Thomas		Pinkney	University of Birmingham
Terrence		Quinn	University of Glasgow
**Data analysis advisor**			
Peter		Nightingale	University Hospitals Birmingham NHS Foundation Trust
**Regional leads**			
Benjamin		Jelley	Cardiff and Vale University Health Board
Victoria		Gaunt	Gloucestershire Hospitals NHS Foundation Trust
Mary		Ni Lochlainn	King’s College London
Kumudhini		Giridharan	Maidstone & Tunbridge Wells NHS Trust
Sarah		Richardson	Newcastle University
Mustafa		Alsahab	Oxford University Hospitals NHS Foundation Trust
Stephen		Makin	University of Aberdeen
Kelli		Torsney	University of Cambridge
Jane		Masoli	University of Exeter
Lindsay		Ronan	University of Exeter
Jenni	K.	Burton	University of Glasgow
Oliver		Todd	University of Leeds
Joanne		Taylor	University of Manchester
Ruth		Willott	University of Nottingham
Natalie		Cox	University of Southampton
Roisin		Healy	Western Health and Social Care Trust
**Other collaborators**			
Nedaa		Haddad	Anuerin Bevan University Health Board
Sharan		Ramakrishna	
Zahid		Subhan	
Antonella		Mazzoleni	Barnet, Enfield and Haringey Mental Health Liaison Service
Olga		Nynaes	
Jodie		Crofts	Barnsley District General Hospital
Emily		McNicholas	
Hannah	J.	Robinson	
Thyn		Thyn	
Jonathan		Baillie	Belfast Health and Social Care Trust
William		McKeown	
Caroline		Rice	
Gerrard	W.	Sloan	
Katherine		Williamson	
Yasmeen		Hayat	Bolton NHS Foundation Trust
Vee Han		Lim	
Katie		Millichamp	
Amr		Bazaraa	Bradford Teaching Hospitals NHS Foundation Trust
Angharad		Chilton	
Alexander		Harbinson	
John		Headlam	
Elisabeth		Hunter	
Zainab		Hussain	
Al Wakkass		Mahmood	
Liji		Ng	
Srividya		Sundara	
Felicia		Tan	
Alice		Wheeler	
Sophie		Wright	
Jack	B.G.	Baldwin	Brighton and Sussex University Hospitals NHS Trust
Kate		O'shea	
Ghazal		Hodhody	Buckinghamshire Healthcare NHS Trust
Kara		Mayor	
Riana		Patel	
Shiv		Bhakta	Cambridge University Hospitals
Marie		Goujon	
Adriana		Jakupaj	
James		Dove	Camden & Islington NHS Foundation Trust
Matthew		Kearney	County Durham and Darlington NHS Foundation Trust
Vincent		McCormack	
Kirsty		Moore	
Leo		Pope	
Hussun-Ara		Shah	
Megan	E.	Shaw	
Gemma	M.	Smith	
Ryan		Love	East and North Hertfordshire NHS Trust
Maya		Mukundan	
Muhammad		Shahid	
Ahmad		Alareed	
Clementine		Anderson	
Karen		Beharry	
Ganapathy		Bhat	
Sanojan		Bremakumar	
Laurence		Caines	
Sandra		Darko	
Nisha		Rai	
David		Saliu	
Pedro		Vila De Mucha	
Phillipa		Adams	Gateshead Health NHS Foundation Trust
Helen		McDonald	Gloucestershire Hospitals NHS Foundation Trust
Sam		Mills	
Frances		Parry	
Frances		Rickard	
Stuart		Winearls	
Rinata		Farah	Great Western Hospitals NHS Trust
Robert		Grange	
Fiona		Herbert	
Elizabeth		Lonsdale-Eccles	
Qurrat		Ul Ain	
Hannah		Watson	
Celine		Bultynck	Guy’s & St Thomas’ NHS Foundation Trust
Chiara		Cavaliere	
Cal		Doherty	
Sarah		Evans	
Daniel		Furmedge	
Annabel		Hentall MacCuish	
Esther		Hindley	
Caitlin		Meyer	
Emma		Mullarkey	
Rosamund		Pullen	
Aidan		Ryan	
Dhruv		Sarma	
Elaine		Seymour	
Katharine		Stambollouian	
Darmiga		Thayabaran	
Chenxian		Wu	
George		Peck	Imperial College London Healthcare Trust
Mahrukh		Raza	
Kapil		Sahnan	
Amber		Dhillon	Keele University
Oluwatosin	O.	Abiola	King's College Hospital NHS Foundation Trust
Catherine		Bryant	
Rachael		Bygate	
John		Frewen	
Isabel		Greaves	
Olivia		Morrow	
Simon		Tetlow	
Guy		Tinson	
Aayenah		Yunus	
Simran		Bedi	King's College London
Olivia		Evans	Lewisham & Greenwich NHS Trust
Leeying		Giet	
Taran		Nandra	
Abolfazl		Behbahani	Liverpool University
Saurav		Bhattacharya	Maidstone and Tunbridge Wells NHS Trust
Clare		Hunt	
Rajeev		Mishra	
Louise		Connor	Newcastle University
Jack		Poynton	
Elizabeth		Deacon	Newcastle Upon Tyne Hospitals NHS Foundation Trust
Rory		Durcan	
Emma		Fisken	
Susan	A.	Hall	
Jane		Noble	
Emma	L.	Prendergast	
Ajay		Macharouthu	NHS Ayrshire and Arran
Victoria		Macrae	
Emily		Murphy-Ackroyd	
Emily	C.	Rose	
Nicola		Watt	
Mairi		Blair	NHS Forth Valley
Eilidh	R.	Mackenzie	
Roisin		McCormack	
Sophie	J.	Irwin	NHS Gateshead
Alice		Einarsson	NHS Grampian
Ming		Khor	
Baraa		Alhadadi	NHS Greater Glasgow and Clyde
Ewen		Cameron	
Angela		Campbell	
Eileen		Capek	
Lorna		Christie	
Laura		Connell	
Alan		Cook	
Jordanna		Deosaran	
Marianne		Elliott	
Elizabeth		Evans	
Sarah		Fancourt	
Sarah		Henderson	
Ambreen		Hussain	
Karen		Jones	
Dominic		Kelly	
Catherine	Mary	McErlean	
Hazel		Miller	
Fariha		Naeem	
Caroline		Ostrowski	
Megan		Parkinson	
Fadi		Sami	
Alison		Shepherd	
Lindsay		Whyte	
Emily		Wright	
Eilidh	E.C.	Ferguson	NHS Lanarkshire
Michael		Gallagher	
Heather	J.	McCluskey	
Louise		Beveridge	NHS Tayside
Hollie	A.	Clements	
Jasmine		Hart	
Neil		Henderson	
Su Kwan		Lim	
James	E.	Lucocq	
Alison		McCulloch	
Adam	A.	Murray	
Esther	E.Y.	Ngan	
Philippa	K.	Traill	
Amy		Walter	
Michael	S.J.	Wilson	
Abigail		Wrathall	
Zhi Jiun		Yap	
Hashem		Abu-Arafeh	North Middlesex University Hospital NHS Trust
Ilan		Gluck	
Oliver		Mitchell	
Richard		Robson	
Elizabeth	L.	Sampson	
Arunkumar		Annamalai	North Tees and Hartlepool NHS Foundation Trust
Jamal		Bhatti	
Laura		Briggs	
Debbie		Fraser	
Jonathan		Gui	
Eleanor		Leah	
Natasha		Matthews	
Pryankaran		Mithrakumar	
Mohammad		Moad	
Michael		Sen	
Jacqui		Straughan	
Roxana		Taranu	
Kasim		Usmani	
Ayesha		Aamir	North West Anglia NHS Foundation Trust
Amaka		Achara	
Olugbenro		Akintade	
Elizabeth	J.	Ellis	
Sneha		Gurung	
Chioma		Iwu	
Abdullah	B.	Khalid	
Sejlo		Koshedo	
Shonit		Nagumantry	
Nader		Nashed	
Philip		Nwabufor	
Ijeoma	T.	Obi	
Parrthiepan		Visvaratnam	
Edward		Wu	
Marc		Bertagne	Northern Devon Healthcare NHS Trust
Peter		Jackson	
James		Allen	Oxford University Hospitals NHS Foundation Trust
Harriet		Brown	
Jennifer		Champion	
Natasha		Christodoulides	
Olivia		Handley	Poole Hospital NHS Foundation Trust
Fiona	E.	Macdonald	
Laura	J.	Beeley	Royal Cornwall Hospitals NHS Trust
Victoria		Clayton	
Aaron		Kay	
John		Marshall	
Hannah		Morgan	
George		Naish	
Sarah	L.	Cleaver	Royal Devon & Exeter Hospital NHS Foundation Trust
Jenny		Evans	
Abbie		Morrow	
Raj		Amarnani	Royal Free London NHS Foundation Trust
Khai	L.	Cheah	
Claire		Cushen	
Amy		Enfield-Bance	
Martin		Glasser	
Suriti		Govindji	
Shama		Mani	
Jemma		Gregory	Sandwell and West Birmingham NHS Trust
Puja		Jatti	
Asma		Khan	
Helena		Lee	
Helen		Millner	
Huma		Naqvi	
Emily		Williamson	
Teresa		Harkin	Sherwood Forest Hospitals NHS Foundation Trust
Bushra		Khizar	
Anna		Lewis	
Hannah		Pendleton	
Steve		Rutter	
Rohan		Ahmed	Shrewsbury and Telford Hospital Trust
Farah		Coffey	
Waleed		Faheem	
Beth		Hackett	
Elizabeth	A.M.	Holmes	
Ali		Khan	
Zeeshan		Mustafa	
Mark	A.	Bowman	South Eastern Health and Social Care Trust
James		Irvine	
Katherine		Patterson	
Sarah		Horner	South Tees Hospitals NHS Foundation Trust
Ting	W.	Wong	
Christopher		Cairns	South Tyneside and Sunderland NHS Foundation Trust
Kate		Foster	
Alex		Hornsby	
Robbie		Horton	
Laura		Jones	
Rachel		King	
Emily		Lyon	
Ani		Tencheva	
Faye		Wilson	
Lesley	J.	Young	
Eleanor		Giblin	South Warwickshire Foundation Trust
Lleika		Kunaselan	
Olivia		Lowes	
Reema		Menezes	
Abigail		Taylor	
Alex		Timperley	
Rachel		Batho	
Charlotte		Bell	
Sammy		Carter	
Paul		Croft	
Eliza		Griffiths	
Giles		Hall	
William		Hunt	
Holly		Jacques	
Felicity		Leishman	
Seema		Murthy	
Sinead		Quinn	
James		Reid	
Amy		Walker	
Stephanie	A.	Matthews	Southend University Hospital Trust
Ayoub		Behbahani	Southport and Ormskirk Hospital NHS Trust
Martin		Glasser	St George's University Hospital NHS Trust
Ana		Silva Ferreira	Surrey and Sussex Healthcare NHS Trust
Caroline		Ashton	The Princess Alexandra Hospital NHS Trust
Swetha		Byravan	The Royal Wolverhampton NHS Trust
Laura		Cummings	
Sana		Faruq	
Sarah		Jagdeo	
Philip		Thomas	
Karen		Broadhurst	Torbay and South Devon NHS Foundation Trust
Joseph	B.	Wilson	
Helen		Bowden	University College London
Katrin		Hoffman	
Howell	T.	Jones	
Charles		Katz-Summercorn	
Ethan		Khambay	
Lucy		Porter	
James		Speed	
Keziah		Austin	University College London Hospitals NHS Foundation Trust
Farrah		Bahsoon	
Rose		Laud	
Jawad		Ali	University Hospitals Birmingham NHS Trust (Birmingham Heartlands Hospital)
Niall		Fergusson	
Claire		Wilkes	
Laura		Babb	University Hospitals Birmingham NHS Trust (Good Hope Hospital) University Hospitals Birmingham NHS Trust (Good Hope Hospital)
James		Gaywood	
Jessica		Green	
Ada		Kantczak	
Katy	F.	Madden	
Sasha		Porter-Bent	
Moe		Su Su San	
Laxmi		Babar	University Hospitals Birmingham NHS Trust (Queen Elizabeth Hospital Birmingham)
Helen		Chamberlain	
Tamsin		Cricklow	
Alexis		Giles	
Abhishek		Gupta	
Clare		Hughes	
Tammy		Lee	
Anum		Cheema	University Hospitals Birmingham NHS Trust (Solihull Hospital)
Yathu		Matheswaran	
Asiodu		Nneamaka	
Anekea		Ross	
Tarunya		Vedutla	
Theresa	J.	Allain	University Hospitals Bristol NHS Trust
Emily		Bowen	
Julie		Dovey	
Natalie		Gaskell	
Deborah		Scott	
Emma		Stratton	
Miriam		Thake	
Stuart		Bullock	University Hospitals Coventry and Warwickshire NHS Trust
Siobhan		McKay	
Stephanie		Radoja	
Sherif		Abdelbadiee	University Hospitals of Leicester NHS Trust
Samuel		Cohen	
Jane		Giddings	
Christopher	J.	Miller	
Emma		Mumtaz	
Minal	D.	Patel	
Vishnu		Prasad	
Lahiru		Satharasinghe	
Mark		Studley	
Marylin		Browne	University Hospitals of North Midlands NHS Trust
Sabrina	K.	Durrant	
Emma		Jay	
Alex		McQuillan	
Megan		Offer	
Jessel		Varghese	
Alexander		Baron	University Hospitals Plymouth NHS Trust
Christian		Chourot	
Peter		Jackson	
Kimberley		Kirrane	
Helen		Rayner	
Kate		Tantam	
Ebrahiem		Tumi	
Shabnam		Venkat	
Nisha		Aggarwal	University of Birmingham
Emma		Astaire	
Karthik		Basker	
L		Berwick	
Edward		Bilton	
Aimee-Louise		Chamboult	
George		Chapman	
Jasmine		Chevolleau	
Grace		Fenneley	
Shannelle		House	
Nathan		Ingamells	
Emilia		Jewell	
Vickneswaran		Kalyaani	
Mahum		Kiani	
Nagarjun		Konda	
Anusha		Kumar	
Jo		Lai	
Jamie		Large	
Joanna		Livesey	
Zeinab		Majid	
Jack		McCready	
Hannah		Moorey	
Bethan		Morgan	
Kirty		Morrison	
Alice		Mosley	
Adam		Pailing	
Sophie		Pettler	
Shayan		Rashid	
Lucy		Rimmer	
Danielle		Scarlett	
Gurpreet		Sehmi	
Abigail		Smith	
Nina		Smith	
Charles		Sprosen	
Emily		Taylor	
Jemima		Taylor	
Connie		Tse	
Sophie		Turton	
Henry		Vardon	
Jasmine		Virk	
Sarah		Warwicker	
Hannah		Woodman	
Beth	L.	Woodward	
Luke		Wynne	
Leah		Yule	
Asim		Ahmad	University of Bristol
Paapa		Appiah-Odame	
Ciaran		Barlow	
Dorothy		Kuek	
Isabelle		Nicholls	
Emma		Norman	
Wioletta		Pyc	
Ashish		Vasudev	
Lawrence	A.T.	Adams	University of Dundee
Emma		Box	
Chung Sien		Chai	
Darcy	S.	Wilson	
Bogna		Drozdowska	University of Glasgow
Emma		Elliott	
Adam		Stirling	
Martin		Taylor-Rowan	
Hannah		Webb	
Li		Wong	
Ahmed		Abras	Walsall Healthcare NHS Trust
Muhammad		Adam	
Zarah		Amin	
Olivia		Cooper	
Rhianna		Davies	
Wan Idoracaera	C.	Ikhwan	
Georgia	R.	Layton	
Awolkhier		Mohammedseid-Nurhussien	
Sohail		Shakeel	
Hana		Waraich	
Jabed		Ahmed	West Hertfordshire Hospitals NHS Trust
Katie		Ball	
Kwasi		Debrah	
Valerie		Page	
Zhao		Xiao Bei	
Hannah		McCauley	Western Health and Social Care Trust
David		McShane	
Freya		Cooper	Weston Area Health NHS Trust
Natalie		Grundmann	
Michael		Haley	
Andre		Le Poideven	
Sarah	B.	McClelland	
Emily		Moore	
Norman		Pang	
Hannah		Currie	Wye Valley NHS Trust
Jayne		Davies-Morris	
Sarah		Edwards	
Sureena		Janagal	
Rodric		Jenkin	
Polly		Jones	
Gary		Kumbun	
Sarah		Parry	
Bhav		Tyagi	
Janine		Valentine	Yeovil District Hospital NHS Foundation Trust
Saad		Abdullah	York Teaching Hospital NHS Foundation Trust
Emmy		Abu	
Sarah		Ahmad	
Bilquis		Ahmed	
Hamza		Ahmed	
Ana		Andrusca	
Matthew		Ansell	
Thomas		Arkle	
Imola		Bargaoanu	
Charlotte		Chuter	
Katie		Houldershaw	
Jacqueline		Ibanichuka	
Shoaib		Iqbal	
Angela		Kabia	
Ismail		Kadir	
Anjli		Krishan	
Adam		McClean	
Gerry		McGonigal	
Pranav		Mishra	
Gladys		Ofoche	
Anna		Reay	
Simon	M.	Stapley	
Adam		Swietoslawski	
Nihaad		Syed	
Karthika		Velusamy	
Afnan		Wahballa	
James		Wilcockson	

## References

[1] Geriatric Medicine Research Collaborative . Delirium is prevalent in older hospital inpatients and associated with adverse outcomes: results of a prospective multi-centre study on world delirium awareness day. BMC Med 2019; 17: 229. 31837711 10.1186/s12916-019-1458-7 PMC6911703

[2] Shrestha P , Fick DM. Family caregiver's experience of caring for an older adult with delirium: a systematic review. Int J Older People Nurs 2020; 15: e12321. 32374518 10.1111/opn.12321

[3] Davis DH , Muniz Terrera G, Keage H et al. Delirium is a strong risk factor for dementia in the oldest-old: a population-based cohort study. Brain 2012; 135: 2809–16. 22879644 10.1093/brain/aws190 PMC3437024

[4] Welch C , Jackson TA. Can delirium research activity impact on routine delirium recognition? A prospective cohort study. BMJ Open 2018; 8: e023386. 10.1136/bmjopen-2018-023386 PMC6252640 30385447

[5] National Institute for Health and Care Excellence . Delirium: Prevention, Diagnosis and Management 2019. Available from: https://www.nice.org.uk/guidance/cg103 (15 November 2021, date last accessed).

[6] National Insitute for Health and Care Excellence . Delirium in Adults 2014. Available from: https://www.nice.org.uk/guidance/qs63/chapter/Quality-statement-5-Communication-of-diagnosis-to-GPs (15 November 2021, date last accessed).

[7] Bellelli G , Morandi A, Davis DH et al. Validation of the 4AT, a new instrument for rapid delirium screening: a study in 234 hospitalised older people. Age Ageing 2014; 43: 496–502. 24590568 10.1093/ageing/afu021 PMC4066613

[8] Tieges Z , Maclullich AMJ, Anand A et al. Diagnostic accuracy of the 4AT for delirium detection in older adults: systematic review and meta-analysis. Age Ageing 2020; 50: 733–43. 10.1093/ageing/afaa224 PMC8099016 33951145

[9] Richardson SJ , Davis DHJ, Stephan BCM et al. Recurrent delirium over 12 months predicts dementia: results of the delirium and cognitive impact in dementia (DECIDE) study. Age Ageing 2020; 50: 914–20. 10.1093/ageing/afaa244 PMC8099011 33320945

[10] Kripalani S , LeFevre F, Phillips CO, Williams MV, Basaviah P, Baker DW. Deficits in communication and information transfer between hospital-based and primary care physicians: implications for patient safety and continuity of care. JAMA 2007; 297: 831–41. 17327525 10.1001/jama.297.8.831

[11] Ryan DJ , O'Regan NA, Caoimh RÓ et al. Delirium in an adult acute hospital population: predictors, prevalence and detection. BMJ Open 2013; 3: e001772. 10.1136/bmjopen-2012-001772 PMC3549230 23299110

[12] Bellelli G , Morandi A, Di Santo SG et al. "Delirium Day": a nationwide point prevalence study of delirium in older hospitalized patients using an easy standardized diagnostic tool. BMC Med 2016; 14: 106. 27430902 10.1186/s12916-016-0649-8 PMC4950237

[13] Tauro R . Delirium awareness – improving recognition and management through education and use of a care pathway. BMJ Qual Improv Rep 2014; 2: u203195.w1451. 10.1136/bmjquality.u203195.w1451 PMC4663845 26732370

[14] Bauernfreund Y , Butler M, Ragavan S, Sampson EL. TIME to think about delirium: improving detection and management on the acute medical unit. BMJ Open Quality 2018; 7: e000200. 10.1136/bmjoq-2017-000200 PMC6109807 30167472

[15] NHS Digital. Hospital Admitted Patient Care Activity 2019–20, 2020. https://digital.nhs.uk/data-and-information/publications/statistical/hospital-admitted-patient-care-activity/2019-20 (15 November 2021, date last accessed).

[16] Kuhn E , Du X, McGrath K et al. Validation of a consensus method for identifying delirium from hospital records. PLoS One 2014; 9: e111823. 25369057 10.1371/journal.pone.0111823 PMC4219785

[17] Geriatric Medicine Research Collaborative . Retrospective delirium ascertainment from case notes: a retrospective cohort study. BMJ Open 2021; 11: e042440. 10.1136/bmjopen-2020-042440 PMC8166612 34049901

[18] Mcleod A, Sampson L, MacLullich A, Squires C, Vardy E . World Delirium Awareness Day Four Years On: Where has it taken us? British Geriatrics Society (Blog) 2021. https://www.bgs.org.uk/blog/world-delirium-awareness-day-four-years-on-where-has-it-taken-us (15 November 2021, date last accessed).

[19] Socttish Intercollegiate Guidelines Network . Risk Reducation and Management of Delirium: Health Improvement Scotland; 2019. Available from: https://www.sign.ac.uk/media/1423/sign157.pdf (15 November 2021, date last accessed).

[20] Jackson TA , Mac Lullich AMJ, Gladman JRF, Lord JM, Sheehan B. Undiagnosed long-term cognitive impairment in acutely hospitalised older medical patients with delirium: a prospective cohort study. Age Ageing 2016; 45: 493–9. 27076525 10.1093/ageing/afw064

