# Supplementary material for: Improving delirium screening and recognition in UK hospitals: results of a multi-centre quality improvement project
Source: Age Ageing. 2022 Feb 25;51(2):afab243. doi: 10.1093/ageing/afab243 (PMC8876302; doi:10.1093/ageing/afab243)
Supplement: aa-21-0029-File002_afab243 [file aa-21-0029-file002_afab243.docx]

Supplementary material

Improving delirium screening and recognition in UK hospitals: results of a multi-centre quality improvement project

Contents

[Methods 2](#_Toc85804258)

[Context 2](#_Toc85804259)

[Interventions 2](#_Toc85804260)

[Measuring change 3](#_Toc85804261)

[Other variables 5](#_Toc85804262)

[Central data collation 6](#_Toc85804263)

[Statistical analysis 6](#_Toc85804264)

[Results 7](#_Toc85804265)

[Interventions implemented at sites 9](#_Toc85804266)

[Educational interventions: impact on screening and recognition 9](#_Toc85804267)

[Discussion of supplementary results (local site interventions) 14](#_Toc85804268)

[References 15](#_Toc85804269)

# Methods

## Context

The Geriatric Medicine Research Collaborative (GeMRC) is a trainee-led network of clinicians working within geriatric medicine (1, 2), which works collaboratively on projects to improve the care of older adults in hospital. Through regional dissemination of projects, trainees working within acute care trusts across the UK are able to be involved. A central team was responsible for coordinating the project, which was able to draw on quality improvement knowledge and experience from all members. This project took a multidisciplinary approach with involvement of higher specialist trainees in geriatric medicine with consultant geriatrician supervision, junior trainees, medical students, nurses, and allied health professionals. Local site teams were responsible for implementing local changes and collecting data at site.

## Interventions

This quality improvement project used a pragmatic approach to implementing changes at individual sites driven by sharing knowledge and tools at a national scale. Participating sites were invited to share local guidelines, toolkits, and presentations so that these could be adapted for implementation at other sites. These were collected and shared at any time between data collection timepoints, and there was no set time to dissemination. We utilised the network developed through each timepoint of measuring change, as described below, to disseminate knowledge. We collected contact details of all collaborators who participated in data collection at each timepoint to generate a mailing list related to this project. This included dissemination of findings from earlier timepoints and invitations to participate in subsequent data collection timepoints. Regional representatives (at least one/region) were invited to attend central GeMRC virtual meetings, where the project was discussed, to encourage regional dissemination. Otherwise sharing of guidelines and toolkits was reliant on the use of a shared Google Docs folder, which all collaborators were invited to access. Additionally, we used social media and web-based communication to increase knowledge of delirium on a wider non-targeted scale, particularly on World Delirium Awareness Day (WDAD) 2018 and 2019. Importantly, the presentation of results of each of our timepoints of measurement change was dynamic, with presentations of our results at national and international conferences. Sites disseminated their local results in relation to the national results after each data collection timepoint.

## Measuring change

There were three main aspects that were assessed as part of this project in terms of assessing the impact of our interventions across the three separate timepoints of data collection:

1. Screening rates for delirium
2. Recognition rates of delirium
3. Documentation of delirium diagnosis on discharge summaries

The first and last timepoint of data collection were performed prospectively on WDAD – March 14^th^ 2018, March 13^th^ 2019. On these dates, we prospectively assessed newly admitted (unscheduled) patients aged ≥65 years-old to acute care trusts (all specialties, excluding critical care and patients discharged from emergency departments). Our methodology and results of our first timepoint of data collection have been published previously (3). Briefly, we screened all patients using the 4 “A”s Test (4AT) as a one-off assessment (4). Screening and data collection was performed by clinical staff or students working within the hospital site. At least one collaborator at each site had expertise in geriatric medicine and supported the other data collectors. Data collectors did not normally have clinical responsibility for patients screened as part of the study. Inter-rater reliability was not measured, but has previously been shown to be excellent for the 4AT (5). If screening was negative (4AT<4) then they were considered not to have delirium. If screening was positive (4AT≥4), we conducted a further assessment in accordance with the Diagnostic and Statistical Manual of Mental Disorders, Fifth edition (DSM-5) (6) to diagnose delirium. If screening was performed by a student, this further assessment was performed by a qualified doctor. For all patients, we recorded if they had been screened for delirium by the usual care team prior to review as part of the study. In those with confirmed delirium, we also recorded if delirium has been recognised by the usual care team. Subtype of delirium was recorded where possible.

Our second timepoint of data collection used a retrospective methodology (7). This methodology was piloted as part of a previous project and shown to have excellent inter-rater reliability. This included all unscheduled admissions of patients aged ≥65 years-old to acute care trusts on September 14^th^ 2018 (all specialties, excluding critical care and patients discharged from emergency departments). We assessed if patients were screened for delirium within 48 hours of admission. We also recorded if delirium was diagnosed at any point during admission and if this was present on admission (prevalent), or developed during their hospital stay (incident). Where delirium was not diagnosed during admission, data collectors retrospectively assessed clinical notes for evidence of delirium using a validated approach (8), based on DSM-5. Delirium diagnosed retrospectively was considered unrecognised delirium. Subtype was recorded where possible. For all three timepoints, data collectors retrospectively assessed discharge summaries for patients with recognised delirium to assess if the diagnosis had been documented.

## Other variables

In order to ensure that we could account for the effect of other variables upon the effects of screening and recognition we also recorded the 9-point Clinical Frailty Scale (CFS) (9), age, gender, specialty, and dementia status (including undiagnosed probable cases, where there was documentation of pre-existent progressive cognitive impairment affecting the patients daily living prior to admission). At the second and third timepoints, we specifically recorded the grade/profession of screener where screening had been performed. In order to assess for interventions with the most notable positive impacts we recorded site-specific information at each timepoint of data collection including the presences of screening tools within the clerking proforma, local delirium guidelines, local delirium leaflets, geriatric medicine teams embedded within the acute admissions unit, or a local specialist delirium team. This information was collected from a survey distributed to each site at the end of data collection at each timepoint. After timepoints 2 and 3, we also included additional questions for sites about interventions that were implemented at sites between timepoints. This included pre-specified interventions (new/expanded specialist delirium team, revised clerking booklet, expansion of geriatric medicine service within admissions unit, new/revised delirium guidelines, new/revised delirium leaflet, local delirium education, delirium posters displayed in admissions unit) as well as options for sites to specify additional interventions. Further details of the implemented interventions were not obtained.

## Central data collation

De-identified data were collected centrally. At the second and third timepoint, data were uploaded by local site teams to REDCap, which is a secure browser-based web application that enables protected collation of data.

## Statistical analysis

Data were imported into IBM SPSS Statistics Version 26 (IBM Corp, NY). We used chi-squared analysis to assess the significance of differences in screening, recognition, and discharge documentation rates across the three timepoints. We used multivariable logistic regression analysis to assess the comparative odds of screening (by the usual care team prior to screening for audit purposes), recognition, and documentation of delirium on discharge summaries between each timepoint of data collected. Variables included in our multivariable analysis were CFS, age, gender, dementia, and specialty. Subtype was also included in models for recognition and discharge documentation. CFS was analysed as an ordinal scale (1-3,4,5,6,7,8), with 9 as a distinct category (CFS 9 applies to patients who are at high risk of dying but are not otherwise frail, and does not suggest more severe frailty). We also performed logistic regression analysis across all timepoints to assess for the impact of site-specific factors on screening, recognition, and documentation on discharge summaries. We included screening and grade/profession of screener within our recognition model to assess if screening was associated with odds of recognition, and if this was affected by the grade/profession of screener. We descriptively assessed data on interventions that were implemented between rounds. We used forest plots generated using RevMan Version 5.3 (10) to graphically and quantitatively assess the impact of the most commonly implemented interventions upon screening and recognition.

# Results

Table S1 – List of participating hospital sites

| Aberdeen Royal Infirmary |
| --- |
| Addenbrooke's Hospital |
| Alexandra Hospital, Redditch |
| Altnagelvin Hospital |
| Barnet Hospital |
| Barnsley Hospital |
| Belfast City Hospital |
| Birmingham Heartlands Hospital |
| Bradford Hospital |
| Bristol Royal Infirmary |
| Cheltenham General |
| City Hospital, Birmingham |
| City Hospitals Sunderland |
| Derriford Hospital |
| East Surrey Hospital |
| Forth Valley Royal Hospital |
| Glasgow Royal Infirmary |
| Gloucester Royal Hospital |
| Good Hope Hospital |
| Great Western Hospital |
| Hereford County Hospital |
| Hinchingbrooke Hospital |
| Inverclyde Royal Hospital |
| James Cook University Hospital |
| John Radcliffe Hospital |
| King's College Hospital |
| King's Mill Hospital |
| Leicester Royal Infirmary |
| Lister Hospital |
| Maidstone Hospital |
| Newcastle upon tyne hospitals |
| Ninewells Hospital |
| North Devon District Hospital |
| North Middlesex Hospital |
| North Tees and Hartlepool |
| Perth Royal Infirmary |
| Peterborough City Hospital |
| Poole Hospital |
| Princess Royal Hospital, Telford |
| Queen Elizabeth Hospital Birmingham |
| Queen Elizabeth Hospital Gateshead |
| Queen Elizabeth University Hospital Glasgow |
| Queen Elizabeth, The Queen Mother Hospital, Margate |
| Royal Alexandria Hospital, Paisley |
| Royal Bolton Hospital |
| Royal Cornwall Hospital |
| Royal Devon |
| Royal Free Hospital |
| Royal Stoke Hospital |
| Royal Sussex County Hospital |
| Royal Victoria Hospital, Belfast |
| Sandwell General Hopsital |
| Scarborough General Hospital |
| Sheffield Teaching Hospital |
| Solihull Hospital |
| South Tyneside Hospital |
| Southend University Hospital |
| Southport Hospital |
| St Marys Hospital |
| St Thomas' Hospital |
| Stoke Mandeville Hospital |
| The Princess Alexandra Hospital, Harlow |
| Torbay Hospital |
| Ulster Hospital |
| University College Hospital, London |
| University Hospital Ayr |
| University Hospital Crosshouse |
| University Hospital North Durham |
| University Hospital of Coventry and Warwick |
| University Hospital of North Tees |
| University Hospital Wishaw |
| University Hospitals Bristol |
| Walsall Manor Hospital |
| Warwick Hospital |
| Watford General Hospital |
| Weston General Hospital |
| Whittington Hospital |
| Wolverhampton New Cross |
| Woolwich Hospital |
| Yeovil District Hospital |
| York Teaching Hospital |
| Ysbyty Ystrad Fawr |

## Interventions implemented at sites

Table S2 – Interventions implemented locally at individual sites

This information was collected from a survey distributed to all sites that participated in timepoints 2 and 3. Some sites implemented multiple interventions, whereas other sites stated that no specific local interventions were implemented. Data were missing from sites who either only participated in Timepoint 1 of data collection, or did not respond to the site surveys distributed at the end of data collection at Timepoint 2 and/or Timepoint 3.

An admiral nurse is a specialist nurse with specialist expertise in providing support to relatives and carers of people with cognitive impairment. Admiral nurses have traditionally been used in community settings to provide support for relatives and carers of people living with dementia (11); the integration of such nurses within secondary care for patients with delirium is novel. Specific details of how this service was implemented from the one site that introduced this change were not collected.

|  | Implemented between timepoints 1 and 2 – N (%) | Implemented between timepoints 2 and 3 – N (%) |
| --- | --- | --- |
| Education | 26 | 23 |
| Posters | 8 | 8 |
| New/ amended guidelines | 8 | 3 |
| New/amended leaflet | 7 | 0 |
| New delirium team | 2 | 0 |
| Electronic delirium tool/alert | 1 | 1 |
| Delirium tool embedded into clerking proforma | 4 | 3 |
| New geriatric medicine team embedded into admissions unit | 12 | 8 |
| New admiral nurse service | 0 | 1 |
| No specific local interventions | 13 | 7 |
| Missing | 33 | 47 |

### Educational interventions: impact on screening and recognition

As by far the most common intervention that was implemented, a specific analysis of the impact of educational interventions upon screening and recognition was performed. Details of how and when educational interventions were delivered were not collected centrally. Numbers at each individual site for each round were small. Three sites showed individually statistically significant improvements in screening rates following local educational interventions. Overall, there was a close statistical trend towards a positive impact of education interventions upon improving screening for delirium (OR 1.31, CI 0.99 – 1.74) (Figure S1). Considering the effect of education upon recognition, numbers at individual sites for each timepoint were even smaller and no sites showed a statistically significant effect. In this analysis educational interventions did not significantly impact upon delirium recognition overall (Figure S2).


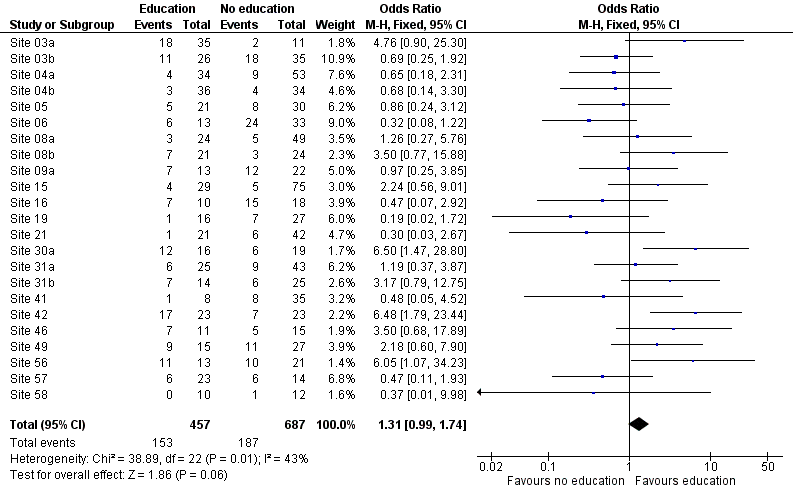


Figure S1 – Forest plot of effect of educational interventions upon delirium screening

This figure demonstrates before and after effects for the likelihood of delirium screening before and after the implementation of educational interventions.

Each individual box shown represents a site with interventions implemented between two timepoints. Where the same intervention type was implemented at a site between more than one timepoint, these are referred to as “a” and “b”, where “a” refers to interventions implemented between Timepoint 1 and 2, and “b” refers to interventions implemented between Timepoint 2 and 3. In this forest plot, “events” refers to the raw number of patients who were screened by the usual care team at site, and “total” refers to the raw number of patients for whom data was collected. “No education” refers to the rates prior to the implementation of the intervention, and “education” refers to the rates after implementation of the intervention. Each box on the forest plot represents the derived odds ratio, with lines denoting the 95% confidence intervals.


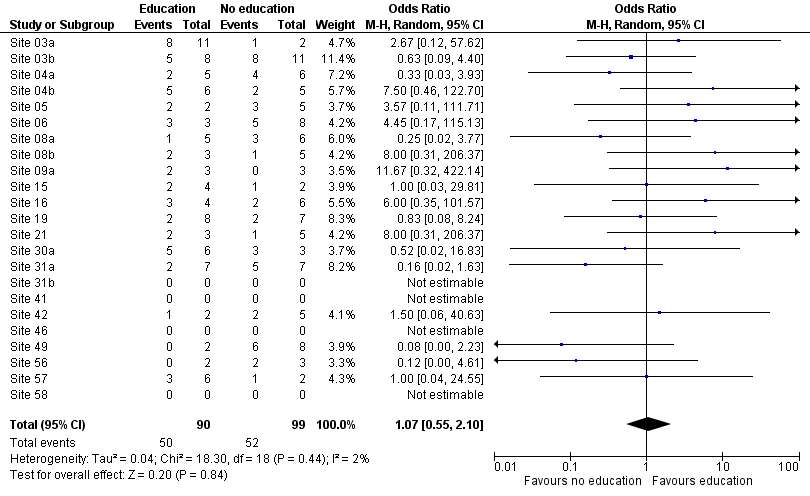


Figure S2 – Forest plot of effect of educational interventions upon recognition of delirium

This figure demonstrates before and after effects for the likelihood of delirium recognition before and after the implementation of educational interventions.

Each individual box shown represents a site with interventions implemented between two timepoints. Where the same intervention type was implemented at a site between more than one timepoint, these are referred to as “a” and “b”, where “a” refers to interventions implemented between Timepoint 1 and 2, and “b” refers to interventions implemented between Timepoint 2 and 3. In this forest plot, “events” refers to the raw number of patients who were recognised as having delirium by the usual care team at site, and “total” refers to the raw number of patients for whom data was collected. “No education” refers to the rates prior to the implementation of the intervention, and “education” refers to the rates after implementation of the intervention. Each box on the forest plot represents the derived odds ratio, with lines denoting the 95% confidence intervals.

Table S3 – Logistic regression for odds of delirium screening

|  | | Beta | SE | Wald | df | OR | 95% CI | | p value |
| --- | --- | --- | --- | --- | --- | --- | --- | --- | --- |
|  |  |  |  |  |  |  | Lower | Upper |  |
| Age | | 0.03 | 0.01 | 25.90 | 1 | 1.03 | 1.02 | 1.04 | <0.001 |
| Gender | | 0.15 | 0.09 | 2.79 | 1 | 1.12 | 0.97 | 1.40 | 0.095 |
| Frailty  (c.f. CFS 1-3) |  |  |  | 13.16 | 5 |  |  |  | 0.022 |
|  | 4 | -0.09 | 0.14 | 0.38 | 1 | 0.92 | 0.69 | 1.21 | 0.538 |
|  | 5 | 0.29 | 0.14 | 4.27 | 1 | 1.33 | 1.01 | 1.75 | 0.039 |
|  | 6 | 0.27 | 0.14 | 3.74 | 1 | 1.31 | 1.00 | 1.71 | 0.053 |
|  | 7 | <0.01 | 0.16 | <0.01 | 1 | 1.00 | 0.73 | 1.38 | 0.992 |
|  | 8 | -0.43 | 0.36 | 1.39 | 1 | 0.65 | 0.32 | 1.33 | 0.238 |
|  | 9 | -0.53 | 0.68 | 0.62 | 1 | 0.59 | 0.16 | 2.21 | 0.430 |
| Dementia | | 0.24 | 0.12 | 4.17 | 1 | 1.28 | 1.01 | 1.61 | 0.041 |
| Specialty (c.f. Acute medicine) |  |  |  | 22.69 | 6 |  |  |  | 0.001 |
|  | Geriatric medicine | 0.19 | 0.12 | 2.41 | 1 | 1.21 | 0.95 | 1.55 | 0.120 |
|  | Other medicine | -0.08 | 0.25 | 0.09 | 1 | 0.93 | 0.57 | 1.52 | 0.766 |
|  | Stroke | -0.23 | 0.12 | 3.74 | 1 | 0.79 | 0.62 | 1.00 | 0.053 |
|  | Other surgery | -0.58 | 0.29 | 4.04 | 1 | 0.56 | 0.32 | 0.99 | 0.044 |
|  | General surgery | -0.70 | 0.23 | 9.52 | 1 | 0.50 | 0.32 | 0.77 | 0.002 |
|  | Orthopaedic surgery | -0.02 | 0.18 | 0.01 | 1 | 0.99 | 0.69 | 1.40 | 0.935 |
| Delirium team | | 0.56 | 0.11 | 25.03 | 1 | 1.75 | 1.41 | 2.18 | <0.001 |
| Clerking tool | | -0.21 | 0.11 | 3.73 | 1 | 0.81 | 0.66 | 1.00 | 0.053 |
| Geriatric medicine within admissions unit | | 0.08 | 0.10 | 0.60 | 1 | 1.08 | 0.89 | 1.31 | 0.439 |
| Delirium guidelines | | -0.33 | 0.13 | 6.47 | 1 | 0.72 | 0.56 | 0.93 | 0.011 |
| Delirium leaflet | | -0.05 | 0.10 | 0.23 | 1 | 0.96 | 0.79 | 1.15 | 0.629 |

Table S4 – Logistic regression for odds of delirium recognition

|  | | Beta | SE | Wald | df | OR | 95% CI | | p value |
| --- | --- | --- | --- | --- | --- | --- | --- | --- | --- |
|  |  |  |  |  |  |  | Lower | Upper |  |
| Screening | | 1.56 | 0.24 | 43.12 | 1 | 4.75 | 2.98 | 7.56 | <0.001 |
| Grade of screener | |  |  | 2.16 | 3 |  |  |  | 0.540 |
| Age | | 0.01 | 0.02 | 0.13 | 1 | 1.01 | 0.97 | 1.04 | 0.721 |
| Gender | | 0.17 | 0.24 | 0.51 | 1 | 1.18 | 0.75 | 1.88 | 0.475 |
| Frailty  (c.f. CFS 1-3) |  |  |  | 6.90 | 5 |  |  |  | 0.229 |
|  | 4 | -0.48 | 0.60 | 0.63 | 1 | 0.62 | 0.19 | 2.01 | 0.429 |
|  | 5 | -0.73 | 0.56 | 1.70 | 1 | 0.48 | 0.16 | 1.44 | 0.193 |
|  | 6 | -1.11 | 0.55 | 4.08 | 1 | 0.33 | 0.11 | 0.97 | 0.043 |
|  | 7 | -1.12 | 0.56 | 4.03 | 1 | 0.33 | 0.11 | 0.97 | 0.045 |
|  | 8 | -1.41 | 0.73 | 3.71 | 1 | 0.24 | 0.06 | 1.03 | 0.054 |
|  | 9 | -0.89 | 2.03 | 0.19 | 1 | 0.41 | 0.01 | 21.79 | 0.660 |
| Dementia | | 0.55 | 0.25 | 4.77 | 1 | 1.73 | 1.06 | 2.84 | 0.029 |
| Subtype | |  |  | 4.81 | 3 |  |  |  | 0.186 |
| Specialty (c.f. Acute medicine) | *Group level* |  |  | 20.29 | 6 |  |  |  | 0.002 |
|  | Geriatric medicine | 0.42 | 0.28 | 2.34 | 1 | 1.53 | 0.89 | 2.63 | 0.127 |
|  | Other medicine | -0.21 | 1.02 | 0.04 | 1 | 0.81 | 0.11 | 6.02 | 0.840 |
|  | Stroke | -0.43 | 0.33 | 1.67 | 1 | 0.65 | 0.34 | 1.25 | 0.197 |
|  | Other surgery | 22.56 | >9999 | <0.01 | 1 | >9999 | <0.01 | >9999 | 1.000 |
|  | General surgery | -2.15 | 0.82 | 6.86 | 1 | 0.11 | 0.02 | 0.58 | 0.009 |
|  | Orthopaedic surgery | -1.31 | 0.55 | 5.74 | 1 | 0.27 | 0.09 | 0.79 | 0.017 |
| Delirium team | | -0.28 | 0.31 | 0.79 | 1 | 0.76 | 0.41 | 1.39 | 0.373 |
| Clerking tool | | -0.13 | 0.28 | 0.23 | 1 | 0.88 | 0.51 | 1.51 | 0.635 |
| Geriatric medicine within admissions unit | | 0.58 | 0.25 | 5.21 | 1 | 1.78 | 1.09 | 2.92 | 0.022 |
| Delirium guidelines | | -0.54 | 0.32 | 2.80 | 1 | 0.58 | 0.31 | 1.10 | 0.094 |
| Delirium leaflet | | 0.33 | 0.25 | 1.66 | 1 | 1.38 | 0.85 | 2.27 | 0.197 |

Table S5 – Logistic regression for odds of documentation on discharge summaries

|  | Beta | SE | Wald | df | OR | 95% CI | | p value |
| --- | --- | --- | --- | --- | --- | --- | --- | --- |
|  |  |  |  |  |  | Lower | Upper |  |
| Age | 0.02 | 0.02 | 1.05 | 1 | 1.02 | 0.98 | 1.06 | 0.307 |
| Gender | -0.18 | 0.28 | 0.40 | 1 | 0.84 | 0.49 | 1.44 | 0.525 |
| Frailty (c.f. CFS 1-3) |  |  | 4.428 | 5 |  |  |  | 0.490 |
| Dementia | 0.76 | 0.28 | 7.19 | 1 | 2.13 | 1.23 | 3.71 | 0.007 |
| Subtype |  |  | 3.63 | 3 |  |  |  | 0.304 |
| Specialty (c.f. Acute medicine) |  |  | 9.75 | 6 |  |  |  | 0.135 |
| Delirium team | 0.31 | 0.35 | 0.78 | 1 | 1.36 | 0.69 | 2.71 | 0.377 |
| Clerking tool | -0.38 | 0.32 | 1.40 | 1 | 0.68 | 0.36 | 1.29 | 0.237 |
| Geriatric medicine within admissions unit | 0.30 | 0.35 | 0.78 | 1 | 1.35 | 0.76 | 2.42 | 0.306 |
| Delirium guidelines | 0.34 | 0.40 | 0.72 | 1 | 1.40 | 0.64 | 3.04 | 0.397 |
| Delirium leaflet | -0.25 | 0.30 | 0.67 | 1 | 0.78 | 0.43 | 1.42 | 0.414 |

# Discussion of supplementary results (local site interventions)

There were a variety of local interventions implemented between timepoints. In many regards, this is typical of any quality improvement project, where drivers are around the improved outcomes, rather than demonstrating cause and effect in the same regards as a randomised controlled trial. There is some suggestion that educational interventions may be effective at improving screening, although this did not reach statistical significance. Details of educational interventions themselves were not collected, and these were likely to have been very heterogeneous. Educational interventions are unlikely to be fully effective by themselves, without sustainable changes in culture and practice (12). Educational programmes should include all multidisciplinary team members, to ensure vigilance in recognition of delirium (13). Interprofessional learning has the potential to enable team members to learn from each other as different professionals may have encountered different signs to prompt them towards a diagnosis of delirium not commonly considered by others. Rates of screening and recognition of delirium were lower amongst patients admitted to surgical specialties; we did not record if sites had an embedded geriatric medicine surgical liaison service at the time of the study.

# References

1. Geriatric Medicine Research Collaborative. Using Social Media and Web-Based Networking in Collaborative Research: Protocol for the Geriatric Medicine Research Collaborative. JMIR research protocols. 2018;7(10):e179.

2. Welch C, Geriatric Medicine Research Collaborative. Growing research in geriatric medicine: a trainee perspective. Age and ageing. 2020;49(5):733-7.

3. Geriatric Medicine Research Collaborative. Delirium is prevalent in older hospital inpatients and associated with adverse outcomes: results of a prospective multi-centre study on World Delirium Awareness Day. BMC Medicine. 2019;17(1):229.

4. Bellelli G, Morandi A, Davis DH, Mazzola P, Turco R, Gentile S, et al. Validation of the 4AT, a new instrument for rapid delirium screening: a study in 234 hospitalised older people. Age and ageing. 2014;43(4):496-502.

5. Kuladee S, Prachason T. Development and validation of the Thai version of the 4 'A's Test for delirium screening in hospitalized elderly patients with acute medical illnesses. Neuropsychiatr Dis Treat. 2016;12:437-43.

6. American Psychiatric Association. Diagnostic and Statistical Manual of Mental Disorders. 5 ed2013.

7. Geriatric Medicine Research Collaborative. Retrospective delirium ascertainment from case notes: a retrospective cohort study. BMJ Open. 2021;11(5):e042440.

8. Kuhn E, Du X, McGrath K, Coveney S, O'Regan N, Richardson S, et al. Validation of a Consensus Method for Identifying Delirium from Hospital Records. PLOS ONE. 2014;9(11):e111823.

9. Pulok MH, Theou O, van der Valk AM, Rockwood K. The role of illness acuity on the association between frailty and mortality in emergency department patients referred to internal medicine. Age and ageing. 2020.

10. The Cochrane Collaboration. Review Manager (RevMan). 2014.

11. Dementia UK. What is an Admiral Nurse and how can they help? 2021 [Available from: <https://www.dementiauk.org/get-support/admiral-nursing/>.

12. Wand APF. Evaluating the effectiveness of educational interventions to prevent delirium. Australasian Journal on Ageing. 2011;30(4):175-85.

13. Aparanji K, Kulkarni S, Metzke M, Schmudde Y, White P, Jaeger C. Quality improvement of delirium status communication and documentation for intensive care unit patients during daily multidisciplinary rounds. BMJ Open Quality. 2018;7(2):e000239.
